# Supplementary material for: Effective Reconstruction of Expectation Values from Ab Initio Quantum Embedding
Source: J Chem Theory Comput. 2023 May 8;19(10):2769–91. doi: 10.1021/acs.jctc.2c01063 (PMC10210252; doi:10.1021/acs.jctc.2c01063)
Supplement: Supplementary file 1 — ct2c01063_si_001.pdf [file ct2c01063_si_001.pdf]

Supplementary Information for 'On the effective reconstruction of expectation values from ab initio quantum embedding'

Max Nusspickel, Basil Ibrahim, and George H. Booth[a)]
Department of Physics, King's College London, Strand, London WC2R 2LS, U.K.

In this SI, we provide example inputs for the `Vayesta` code v1.0.0 (publicly available via https://github.com/BoothGroup/Vayesta) for the generation of a number of representative results from this work.

Input for Chlorine dimer results of Fig. 1, providing all energy functionals described

```
import numpy as np
import pyscf
import pyscf.gto
import pyscf.scf
import pyscf.fci
import vayesta
import vayesta.ewf


optimize_cpt = False
dm0 = None
for d in np.arange(1.0, 5.001, 0.1):
    # --- Setup molecule with PySCF:
    mol = pyscf.gto.Mole()
    mol.atom = "Cl 0 0 0 ; Cl 0 0 %f" % d
    mol.basis = 'STO-6G'
    mol.output = 'pyscf.txt'
    mol.build()

    # --- Obtain Hartree-Fock solution with PySCF:
    mf = pyscf.scf.RHF(mol)
    mf.conv_tol = 1e-10
    mf.conv_tol_grad = 1e-8
    mf.kernel(dm0=dm0)
    dm0 = mf.make_rdm1()

    # --- Reference FCI
    fci = pyscf.fci.FCI(mf)
    fci.kernel()
    e_fci = fci.e_tot

    # --- Embedded CCSD using DMET bath orbitals only (note CCSD is exact for two-hole clusters):
    emb = vayesta.ewf.EWF(mf, solver='CCSD', bath_options=dict(bathtype='dmet'))
    # Fragment the system in terms of symmetrically orthogonalized ("Lowdin") orbitals (SAO)
    # and add two atomic fragments, corresponding to the atoms with indices 0 and 1:
    with emb.sao_fragmentation() as f:
        f.add_atomic_fragment(0)
        f.add_atomic_fragment(1)
    # Optimize fragment chemical potentials based on the democratically-partitioned 1-DM:
    if optimize_cpt:
        emb.optimize_chempot()
    # Construct bath spaces, clusters, and solve embedding problems:
    emb.kernel()

    # --- Energy functionals based on partitioned density-matrices:
    # Partitioned 2-DM (DMET) energy:
    e_dmet_part_2dm = emb.get_dmet_energy(part_cumulant=False)
    # Partitioned 2-DM cumulant (improved DMET) energy:
    e_dmet_part_cumulant = emb.get_dmet_energy()
```

[a)] Electronic mail: george.booth@kcl.ac.uk

Input for a single molecule in the W4-11 test set of Sec. VI A for a range of bath natural orbital thresholds

```
import numpy as np
import pyscf
import pyscf.gto
import pyscf.scf
import pyscf.cc
import vayesta
import vayesta.ewf

# --- Setup molecule with PySCF:
mol = pyscf.gto.Mole()
mol.atom = [('N', [0.0, 0.0, 1.855199]),
            ('C', [0.0, 0.0, 0.693922]),
            ('C', [0.0, 0.0, -0.693922]),
            ('N', [0.0, 0.0, -1.855199])]
mol.basis = 'cc-pvdz'
mol.output = 'pyscf.txt'
mol.build()

# --- Obtain Hartree-Fock solution with PySCF:
mf = pyscf.scf.RHF(mol)
mf.conv_tol = 1e-10
mf.kernel()

# --- Reference CCSD
cc = pyscf.cc.CCSD(mf)
cc.kernel()
e_cc = cc.e_tot

etas = ['DMET', 1e-3, 1e-4, 1e-5, 1e-6]
for eta in etas:
    # --- Embedded CCSD using BNO bath orbitals:
    if eta == 'DMET':
        emb = vayesta.ewf.EWF(mf, solver='CCSD', bath_options=dict(bathtype='dmet'))
    else:
        emb = vayesta.ewf.EWF(mf, solver='CCSD', bath_options=dict(bathtype='mp2', threshold=eta))
    # Fragment the system in terms of intrinsic atomic orbitas (IAOs) with one fragment for each atom:
    with emb.iao_fragmentation() as f:
        f.add_all_atomic_fragments()
    # Construct bath spaces, clusters, and solve embedding problems:
    emb.kernel()

    # Partitioned WF, linear functional, energy:
    e_wf = emb.get_wf_energy()
    # Partitioned WF, density-matrix functional:
    e_dm = emb.get_dm_energy(dm2='global-wf')
    # Partitioned WF, density-matrix functional, with in-cluster approximation of cumulant:
    e_dm_incluster_cumulant = emb.get_dm_energy()

    eta_string = '%.2e'%eta if not eta=='DMET' else ' DMET '
    with open('energies.txt', 'a') as f:
        f.write('%6s  %16.8f  %16.8f  %16.8f  %16.8f\n' % (
                eta_string, e_cc, e_wf, e_dm, e_dm_incluster_cumulant))
```

Input for the periodic diamond cell calculations of Sec. VI B

```
import numpy as np

import pyscf
import pyscf.pbc
```

```
import vayesta
import vayesta.ewf
import vayesta.misc.counterpoise

a = 6.7  # Lattice paramter
Nk = 5    # k-grid / supercell size
etas = [1.e-4, 1.e-5, 1.e-6, 1.e-7] # BNO thresholds

# Build cell
cell = pyscf.pbc.gto.Cell()
cell.unit = 'B'
cell.atom = ['C 0.0 0.0 0.0', 'C %f %f %f' % (a/4, a/4, a/4)]
cell.a = np.asarray([[a/2, a/2, 0],
                     [0, a/2, a/2],
                     [a/2, 0, a/2]])
cell.basis = 'cc-pvtz'
cell.output = 'C_N=%d_a=%.3f.log' % (Nk, a)
cell.max_memory = 182345 # Availible RAM in MB
cell.precision = 1e-12
cell.verbose = 8
cell.build()

# Hartree Fock with Nk x Nk x Nk k-points
kmesh = [Nk, Nk, Nk]
# Only sample the irreducible Brillouin zone
kpts = cell.make_kpts(kmesh, space_group_symmetry=True, time_reversal_symmetry=True, symmorphic=True)
kmf = pyscf.pbc.scf.KRHF(cell, kpts)
print("Running RHF")
# Use range-separated density fitting for two electron integrals
kmf = kmf.rs_density_fit(auxbasis='cc-pvtz-ri')
kmf.kernel()
kmf = kmf.to_khf()
emf = kmf.e_tot
emb_es = [[],[]] # Store the linear and DM energies for each eta value

# Embedded CCSD for a range of BNO thresholds
print("Running Vayesta")
emb = vayesta.ewf.EWF(kmf, solver="CCSD", bno_threshold=1e-4, solve_lambda=True)
# Use symmetry information of the cell to reduce calculations
emb.symmetry.add_rotation(order=2, axis=[1,0,-1], center=[1/8,1/8,1/8], unit='latvec')
with emb.iao_fragmentation() as f:
    f.add_atomic_fragment(0)
for j, eta in enumerate(etas):
    # Loop through bath threshold values
    vayesta.new_log("vayesta-N=%d_a=%.3f_eta=%.2e.log" % (Nk, a, eta))
    # It can save resources to just update the bath options, rather than running new calculations
    emb.change_options(bath_options=dict(threshold=eta))
    emb.reset(reset_bath=False)
    emb.kernel()
    emb_es[0].append(emb.e_corr + emf)              # Linear energy functional
    emb_es[1].append(emb.get_dm_energy() + emf)     # Partitioned WF with in-cluster cumulant

# OPTIONAL: Repeat calculations for single atom and single atom in presence of all basis functions,
# to estimate BSSE correction
print("Calculating BSSE Correction")
emb_bsse = []
for rmax in [False, np.inf]:
    bsse = []
    # Remove periodic boundary conditions from cell, include basis functions for 3x3x3 supercell
    cell = vayesta.misc.counterpoise.make_cp_mol(cell, 0, rmax, nimages=1, unit='B')
    # Run molecular Hartree Fock
    kmf = pyscf.scf.RHF(cell)
    kmf = kmf.density_fit(auxbasis='cc-pvtz-ri')
    kmf.kernel()
    emf = kmf.e_tot

    # Calculate embedded CCSD contribution for a range of BNO thresholds
    emb = vayesta.ewf.EWF(kmf, solver="CCSD", bno_threshold=1e-4, solve_lambda=True)
    with emb.iao_fragmentation() as f:
        f.add_atomic_fragment(0)
```

```
    for j, eta in enumerate(etas):
        vayesta.new_log("vayesta-N=%d_a=%.3f_eta=%.2e.log" % (Nk, a, eta))
        emb.change_options(bath_options=dict(threshold=eta))
        emb.reset(reset_bath=False)
        emb.kernel()
        bsse.append(emf + emb.e_corr)
        bsse.append(emf + emb.get_dm_energy())
    emb_bsse.append(bsse)

emb_es = np.array(emb_es)
emb_bsse = np.array(emb_bsse)
# Add BSSE correction (factor of 2 due to two atoms per unit cell)
es = emb_es + 2*(emb_bsse[0] - emb_bsse[1])
print("shape=%s"%str(es.shape))
with open('output.txt', 'w') as f:
    for i, eta in enumerate(etas):
        f.write("%.2e   %16.8f  %16.8f\n"%(eta, es[0+2*i], es[1+2*i]))
```

Input for generation of non-local spin-spin correlation functions of Sec. VI C

```
import numpy as np
import pyscf
import pyscf.gto
import pyscf.scf
import vayesta
import vayesta.ewf


# --- Setup propyl radical with PySCF
mol = pyscf.gto.Mole()
mol.atom = """
C1       1.3069720      -0.2971550      -0.0288580
C2       0.0763510       0.5643640       0.0457890
C3      -1.2272860      -0.2475040      -0.0331080
H4       2.2845250       0.1356880      -0.2543030
H5       1.2725090      -1.3447380       0.2836320
H6       0.0816430       1.1471200       0.9908800
H7       0.1017350       1.3160270      -0.7643650
H8      -1.2863970      -0.9717310       0.7967330
H9      -1.2802440      -0.8104350      -0.9795620
H10     -2.1099900       0.4098420       0.0240440
"""
mol.spin = 1
mol.basis = 'cc-pVTZ'
mol.output = 'pyscf.txt'
mol.build()

# --- Solve unrestricted Hartree-Fock with PySCF
mf = pyscf.scf.UHF(mol)
mf.kernel()
assert mf.converged

# --- List of all atoms A and B, for which <Sz(A)Sz(B)> is calculated:
atoms_a = [0]
atoms_b = list(range(mol.natm))
atoms = [atoms_a, atoms_b]

for eta in [np.inf, 1e-4, 1e-5, 1e-6, 1e-7, 1e-8, -np.inf]:

    emb = vayesta.ewf.EWF(mf, bath_options=dict(threshold=eta))
    # Use IAO+PAO fragmentation. This is less efficient for wave function partitioning, but will allow for a direct
    # comparison of partioned density-matrix and partitioned wave function functionals:
    with emb.iaopao_fragmentation() as f:
        f.add_all_atomic_fragments()
    emb.kernel()
```

```python
    # --- Sz^2 from democratically-partitioned 2-DM:
    dm1 = emb.make_rdm1_demo()
    dm2 = emb.make_rdm2_demo(part_cumulant=False)
    ssz_part_2dm = emb.get_corrfunc('Sz,Sz', dm1=dm1, dm2=dm2, atoms=atoms)

    # --- Sz^2 from democratically-partitioned 2-DM cumulant:
    dm1 = emb.make_rdm1_demo()
    dm2 = emb.make_rdm2_demo()
    ssz_part_cumulant = emb.get_corrfunc('Sz,Sz', dm1=dm1, dm2=dm2, atoms=atoms)

    # --- Sz^2 from partitioned wave-function DMs (with in-cluster approximation of cumulant):
    ssz_part_wf = emb.get_corrfunc('Sz,Sz', atoms=atoms)
    # This is identical to the following (but avoids building and storing the 2-DM):
    #dm1 = emb.make_rdm1()
    #dm2 = emb.make_rdm2()
    #ssz_part_wf = emb.get_corrfunc('Sz,Sz', dm1=dm1, dm2=dm2, atoms=atoms)

    # --- Write results
    nmean = emb.get_mean_cluster_size()
    results = [ssz_part_2dm, ssz_part_cumulant, ssz_part_wf]
    for atom in atoms_b:
        ssz_atom = [ssz[0,atom] for ssz in results]
        with open('ssz-0-%d.txt' % atom, 'a') as f:
            fmt = '%3d  ' + (len(ssz_atom)*'  %.8e')  + '\n'
            f.write(fmt % (nmean, *ssz_atom))
```
